# Supplementary material for: MetaRibo-Seq measures translation in microbiomes
Source: Nat Commun. 2020 Jun 29;11:3268. doi: 10.1038/s41467-020-17081-z (PMC7324362; doi:10.1038/s41467-020-17081-z)
Supplement: Supplementary file 10 — Supplementary Data 7 [file 41467_2020_17081_MOESM10_ESM.zip › File2/Confidence_High_Taxonomy/161088_out.krona.html]

Javascript must be enabled to view this page.

members
magnitude
magnitudeUnassigned
count
unassigned
taxon
rank

161088\_out

2

superkingdom
2
2

1239
2
phylum

526524
2
class

526525
2
order

family
2
128827

genus
1505663
1


SRS146888\_contig\_number\_13497
species
1
1522


SRS017521\_contig\_number\_contig-100\_7910.234223
469614
1
species
